# Atypical Cerebral Manifestations of Disseminated *Mycobacterium tuberculosis*

**DOI:** 10.3389/fneur.2017.00462

**Published:** 2017-09-21

**Authors:** Ji Hye Hwang, Kyung Mi Lee, Ji Eun Park, Hyug-Gi Kim, Eui Jong Kim, Woo Suk Choi, Na Rae Yang

**Affiliations:** ^1^Department of Radiology, Kyung Hee University College of Medicine, Kyung Hee University Hospital, Seoul, South Korea; ^2^Department of Neurosurgery, Mokdong Hospital, Ewha Womans University School of Medicine, Seoul, South Korea

**Keywords:** tuberculous meningitis, tuberculoma, atypical tuberculosis meningitis, post-contrast fluid-attenuated inversion recovery, disseminated tuberculosis

## Abstract

**Background:**

We investigated the patterns of cerebral manifestations in patients with underlying pulmonary or extrapulmonary tuberculosis or disseminated tuberculosis.

**Materials and methods:**

From January 2010 to September 2016, brain magnetic resonance imaging (MRI) scans were obtained to evaluate cerebral manifestations in patients with underlying pulmonary or extrapulmonary tuberculosis. We also included patients with drug-resistant tuberculosis or disseminated tuberculosis. MRI findings of tuberculous meningitis and tuberculoma were classified as typical; other MRI findings were classified as atypical. Demographic data, risk factors, and drug regimens were collected and analyzed.

**Results:**

Twenty-two patients were diagnosed with cerebral tuberculosis. Cerebral tuberculosis was due to hematogenous spread from pulmonary tuberculosis (10 patients), spinal tuberculosis (8 patients), disseminated tuberculosis (3 patients), and unknown causes (1 patient). There were six patients with typical MRI findings (three patients with typical meningitis involving the basal cistern and supratentorium, one patient with tuberculomas, and two patients with both) and seven patients with atypical MRI findings [five patients with evidence of early meningitis, such as high signal intensity on fluid-attenuated inversion recovery (FLAIR) along the cerebellar folia, and two patients with only hydrocephalus].

**Conclusion:**

Besides the typical sites of meningeal involvement, overlooked findings such as FLAIR abnormalities along the cerebellar folia or hydrocephalus should be checked for early detection of cerebral tuberculosis and initiation of the appropriate treatment against disseminated tuberculosis.

## Introduction

*Mycobacterium tuberculosis* remains a significant public health problem despite a recent decrease in its global incidence. According to the latest World Health Organization report, the estimated prevalence of tuberculosis is 11 million, and an estimated 3.5% of newly developed tuberculosis cases and 20.5% of previously treated tuberculosis cases are resistant to drugs. Among the cases of newly developed tuberculosis, 0.83 million patients have extrapulmonary tuberculosis, including central nervous system (CNS) tuberculosis.

Although pulmonary tuberculosis accounts for the majority of cases, extrapulmonary tuberculosis also contributes to the burden of the disease, thus requiring specific attention. Extrapulmonary tuberculosis is defined as the occurrence of tuberculosis at sites other than the lungs, such as the lymph nodes, genitourinary tract, pleura, bones and joints, meninges and CNS, peritoneum, and other abdominal organs (1). CNS tuberculosis is considered one of the most devastating types of extrapulmonary tuberculosis because it is associated with a high mortality and a high rate of neurological sequelae (2).

The incidence of CNS tuberculosis is 5–10% of extrapulmonary tuberculosis cases and approximately 1% of all tuberculosis cases (2). The exact incidence of CNS tuberculosis is unknown despite its devastating clinical symptoms. Gupta et al. represented that the brain is less frequently involving organ in miliary tuberculosis (3). Spinal tuberculosis accounts for approximately 50% of the cases of skeletal tuberculosis (4). Of 40 acquired immune deficiency syndrome (AIDS) patients with disseminated tuberculosis, seven had CNS involvement, as revealed by meningeal enhancement or round or lobulated nodes or masses with homogeneous or rim enhancement (5).

Tuberculosis is generally defined as tuberculous infection involving two or more non-contiguous sites, including the blood stream, bone marrow, or miliary tuberculosis itself (6) and is considered a lethal form of tuberculosis due to massive lymphohematogenous spreading. It has been studied to be associated with severe immunocompromised status, especially impaired cell-mediated immunity (3, 7). The exact incidence of disseminated tuberculosis is unknown. Wang et al. reported that of the 3,058 patients with culture-confirmed tuberculosis, 164 (5.4%) had disseminated tuberculosis (8). Shafer et al. reported that miliary tuberculosis occurs in 10% of patients with AIDS and pulmonary tuberculosis, and in 38% of patients with AIDS and extrapulmonary tuberculosis (9). Since clinical manifestations of disseminated tuberculosis are non-specific, timely recognition of sings and symptoms is essential to establish the diagnosis. Delayed diagnosis or misdiagnosis of disseminated tuberculosis can delay the initiation of treatment, leading to various complications and increasing the risk of mortality (6).

Herein, we investigated the pattern of brain infection with diagnosed pulmonary tuberculosis or extrapulmonary tuberculosis, or disseminated tuberculosis. Furthermore, we describe the one of the most overlooked cerebral involving pattern of disseminated tuberculosis in patients with underlying untreated or drug-resistant spinal or pulmonary tuberculosis.

## Materials and Methods

### Patient Selection

From our institutional database of patients who were diagnosed with pulmonary or extrapulmonary tuberculosis between January 2010 and September 2016, we selected those who underwent brain magnetic resonance imaging (MRI) examination. Patients whose symptoms and MRI abnormalities were relieved by anti-tuberculosis treatment were tentatively considered as having brain tuberculosis and finally diagnosed with CNS tuberculosis. Patients without neurologic symptoms were tentatively considered as having brain tuberculosis and finally diagnosed with CNS tuberculosis if positive cerebrospinal fluid study.

Patient demographics and medical history were reviewed from electronic medical records. Age, sex, origin site, HIV infection, and drug resistance were investigated. In addition, multidrug-resistant tuberculosis, defined as tuberculosis infection caused by bacteria that are resistant to at least isoniazid and rifampin, was investigated among patients who had a positive tuberculosis culture and performed a drug susceptibility test.

Most patients were provided standard anti-tuberculosis treatment with isoniazid, rifampin, pyrazinamide, and ethambutol, according to Korean guidelines for tuberculosis treatment.

### Image Acquisition

To correlate brain MRI findings with chest computed tomography (CT) or spine CT/MRI findings, we used the brain MRI scans obtained within 1 week prior to and 3 months after the date of chest CT or spine CT/MRI examination. All brain magnetic resonance (MR) images were reviewed independently on the picture archiving and communication system by two neuroradiologists with 6 and 20 years of experience, who were blinded to the final diagnosis. In our institution, MRI scans are obtained on a 1.5T (GE Healthcare, Genesis-Signa) and 3T (Philips, Achieva) scanner. MRI was performed using spin-echo sequences with T1-weighted images [T1WI; repetition time (TR) 450/echo time (TE) 10–12], T2-weighted images (T2WI; TR 4083/TE 115), and fluid-attenuated inversion recovery (FLAIR; TR 11,000 ms/TE 125 ms) images in the axial, sagittal, and coronal planes, with a slice thickness of 3–5 mm. After intravenous administration of the contrast medium (Gadovist, 0.1 mmol/kg), contrast-enhanced (CE)-T1WI and CE-FLAIR images were obtained in the axial or coronal planes.

### Image Analysis

All brain MR images were evaluated for the presence or absence of the following imaging features associated with typical findings of brain tuberculosis: meningeal enhancement on the basal cistern or supratentorium (tuberculosis meningitis); rim or nodular enhancing masses (tuberculomas); and atypical findings such as hydrocephalus and abnormal FLAIR signal changes at the infratentorium or cranial nerves.

On post-contrast images, meningitis was shown as linear or irregular enhancement along the cerebral meninges. Tuberculomas were classified into two types based on the pathological pattern: non-caseating tuberculomas, which usually show high signal intensity (SI) on T2WI and slightly low SI on T1WI; and caseating tuberculomas, which show iso- to high SI on both T1WI and T2WI, with an iso- to high SI peripheral rim on T2WI.

All brain MR images were initially investigated focusing on the typical findings. If there were unusual findings such as abnormal SI or hydrocephalus, the neuroradiologists reported those findings separately and classified as atypical findings of CNS tuberculosis. For these analyses, we focus on the non-contrast FLAIR and CE-FLAIR sequences because FLAIR high SI along the cerebellar folia and cranial nerves were known as very early findings of meningitis with various causes. Furthermore, the hydrocephalus criterion in this article was based on Evans’ index (10). The calculation of index is by dividing the width of the frontal horns of the lateral ventricles by the maximum biparietal diameter. Evans’ index over 0.30 is considered abnormal: mild, moderate, and severe hydrocephalus were defined based on an Evan’s index of <0.34, 0.35–0.40, and >0.40, respectively (11). We also evaluated the temporal horn in follow-up MRI images. If the patients were proven to have hydrocephalus, change of periventricular parenchyma and type of hydrocephalus were investigated. Communicating hydrocephalus is a type of hydrocephalus with a dilated fourth ventricle. There was no evidence of an obstruction of the intraventricular cerebrospinal fluid pathways, including the fourth ventricular outflow and the cerebral aqueduct.

## Results

### Patient Demographics

Twenty-two patients were tentatively diagnosed with brain tuberculosis and primary tuberculosis: 10 patients had been previously diagnosed with underlying pulmonary tuberculosis, 8 patients had underlying spinal tuberculosis, 3 patients had disseminated tuberculosis, and 1 patient had tuberculosis of unknown origin (Table 1).

**Table 1:** Demographic data and magnetic resonance findings of all subjects.

Patient no.	Origin site	Typical FINDINGS		Atypical findings	Anti-HIV Ab	Tb	CSF	Drug resistance
		Meningitis	Tuberculoma					
1	Lung	–	x	–	–	Positive	–	–
2	Lung	x	x	x	Negative	Positive	–	Sensitive
3	Lung	–	x	–	Negative	Positive	–	Resistant (INH)
4	Lung	x	x	o	Negative	Positive	–	Sensitive
5	Lung	–	x	x	Negative	Positive	–	Sensitive
6	Lung	x	x	o	Negative	Positive	–	Resistant (INH, RFP)
7	Lung	x	x	x	Negative	Positive	–	Resistant (INH, SM)
8	Lung	x	x	o	Negative	Positive	–	Sensitive
9	Lung	x	x	x	Negative	Positive	–	–
10	Lung	o	o (multiple)	x	Negative	Positive	Positive	–
11	Spine	–	x	x	Negative	Negative	–	–
12	Spine	x	x	x	Negative	Negative	–	–
13	Spine	x	x	x	Negative	Negative	–	–
14	Spine	o	x	x	Negative	Positive	–	–
15	Spine	o	x	x	Negative	Positive	–	Sensitive
16	Spine	–	x	x	Negative	Negative	–	–
17	Spine	–	x	x	Negative	Negative	–	–
18	Spine	x	x	x	Negative	Negative	–	–
19	Disseminated	x	o (multiple)	x	Negative	Positive	Positive	Sensitive
20	Disseminated	o	o (multiple)	o	Negative	Positive	Positive	Sensitive
21	Disseminated	x	x	x	Negative	Positive	–	–
22	Unknown	o	x	o	Negative	Positive	Positive	Sensitive

*FLAIR, fluid-attenuated inversion recovery; SI, signal intensity; HIV, human immunodeficiency virus; Ab, antibody; TB, tuberculosis; CSF, cerebrospinal fluid; INH, isoniazid; RFP, rifampin; SM, streptomycin*.

Of the 22 patients, 12 were female: 5 of the 10 patients with pulmonary tuberculosis, 5 of the 8 patients with spinal tuberculosis, 1 of the 3 patients with disseminated tuberculosis, and 1 with primary CNS tuberculosis.

Of the 22 patients, 21 were tested for anti-HIV antibody (Ab) because HIV infection is a major risk factor of tuberculosis. All 21 patients were negative for anti-HIV Ab. Two patients were resistant to tuberculosis drugs. One patient was resistant to both isoniazid and rifampin and was considered a case of multidrug-resistant tuberculosis. One other patient was resistant to isoniazid and streptomycin.

Sixteen patients were positive for AFB staining and culture, tuberculosis polymerase chain reaction testing, and interferon-gamma. Four of the 16 patients were confirmed as having brain tuberculosis in the cerebrospinal fluid study. Another six patients were negative for tuberculosis work-up, but clinically diagnosed and treated according to the tuberculosis criteria.

### Image Interpretation

**Figure 1:**
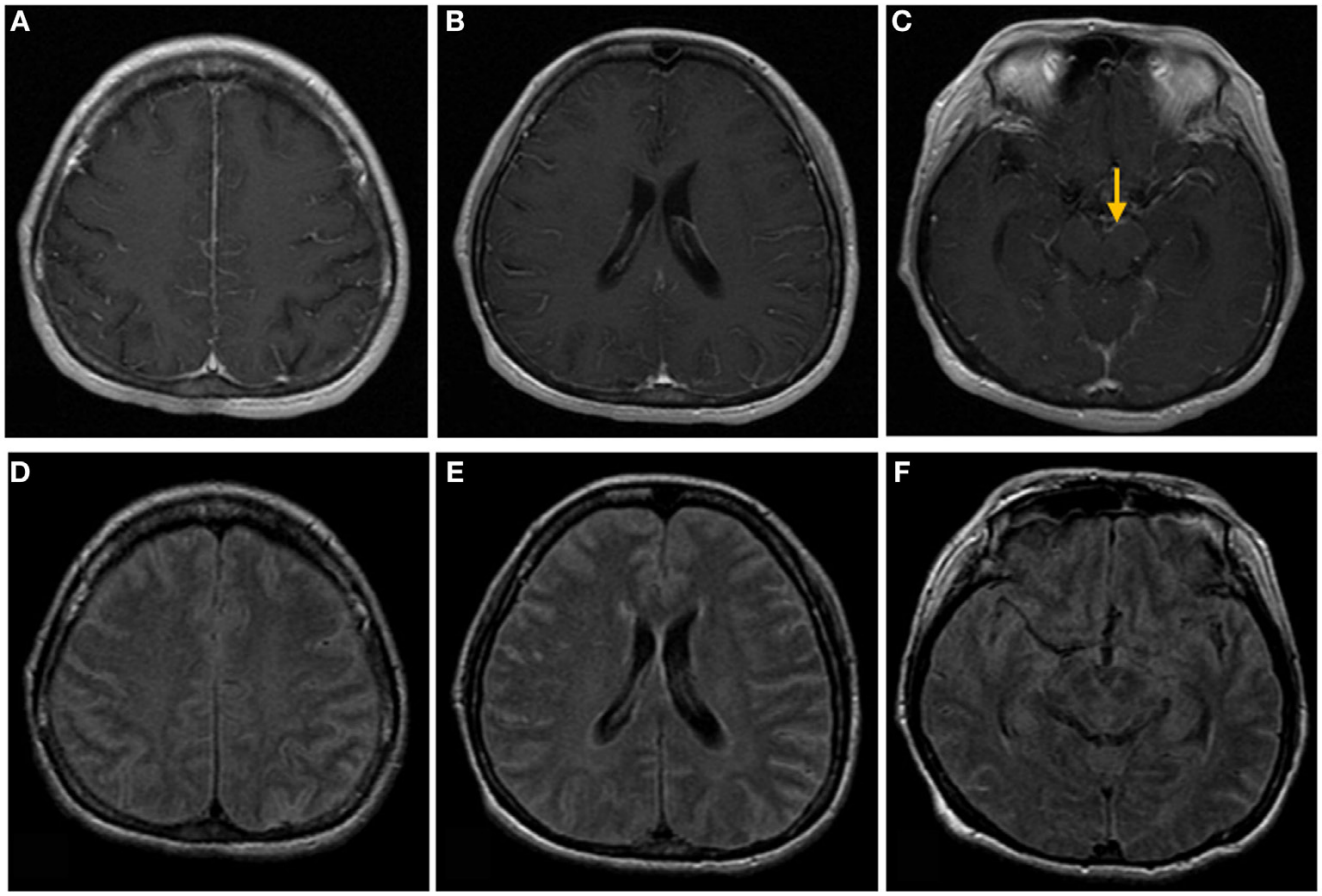
Brain magnetic resonance images of a 50-year-old woman with headache, nausea, and vomiting. **(A–C)** Post-contrast T1-weighted images show linear meningeal enhancement along the cerebral sulci and midbrain surface (arrow). **(D–F)** Fluid-attenuated inversion recovery images show high signal intensities occupying the subarachnoid space, compatible with tuberculosis meningitis.

Of the 22 patients, 5 showed abnormal meningeal enhancement on the basal cistern or supratentorium. The primary origin sites of typical meningitis were as follows: pulmonary tuberculosis (one patient), spinal tuberculosis (two patients), disseminated tuberculosis (one patient), and unknown origin (one patient) (Figure 1).

**Figure 2:**
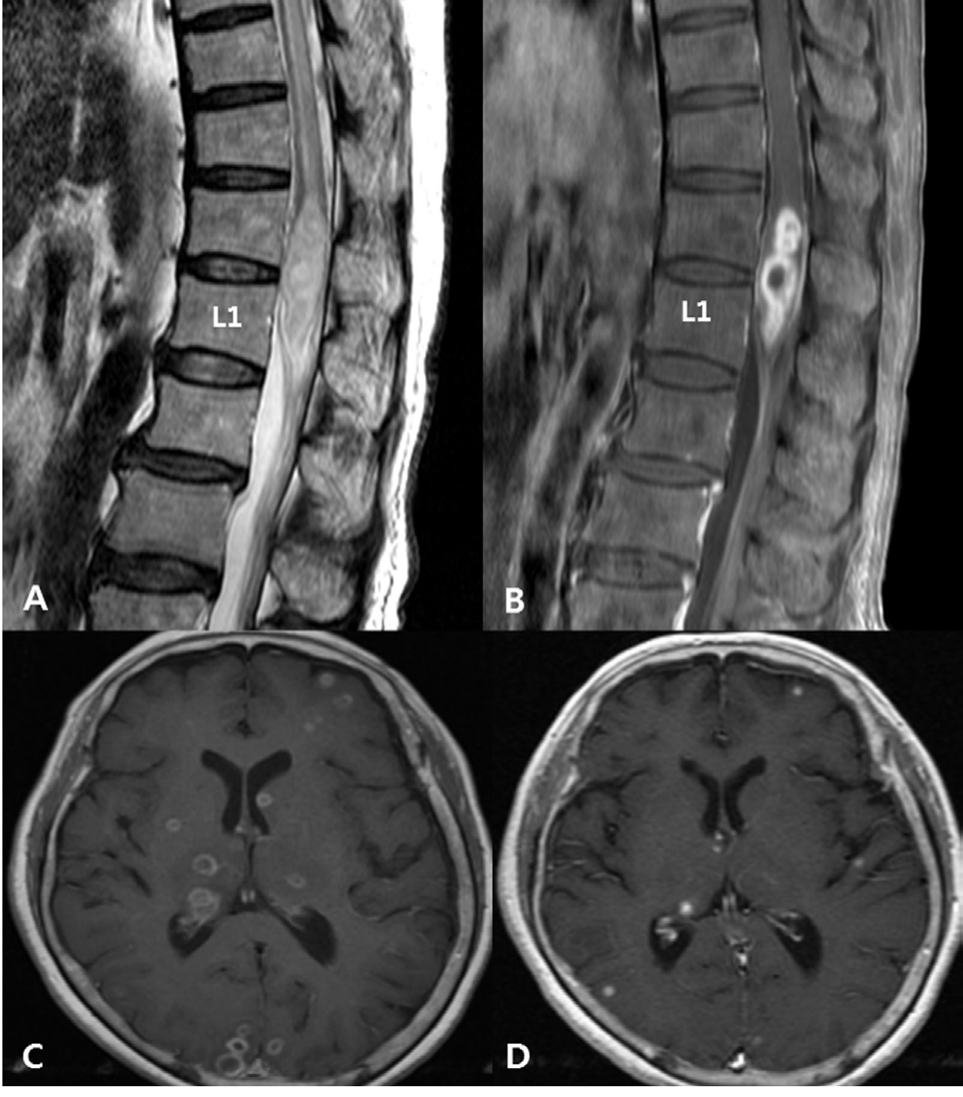
Disseminated tuberculosis in a 72-year-old woman with pain in both lower extremities with underlying active pulmonary tuberculosis. **(A)** Spinal magnetic resonance (MR) T2-weighted images show intermediate to slightly high signal intensities and cord edema at the T7–L1 level. **(B)** Post-contrast T1-weighted images with fat suppression, taken at the same level as the images in panel **(A)**, show rim enhancement at the intramedullary portion of the conus medullaris, suggesting a tuberculous abscess. **(C)** Initial post-contrast T1-weighted brain MR image shows multiple tuberculomas with rim enhancing pattern. **(D)** Follow-up MR image shows regression of tuberculomas after a 1-month anti-tuberculosis treatment.

**Figure 3:**
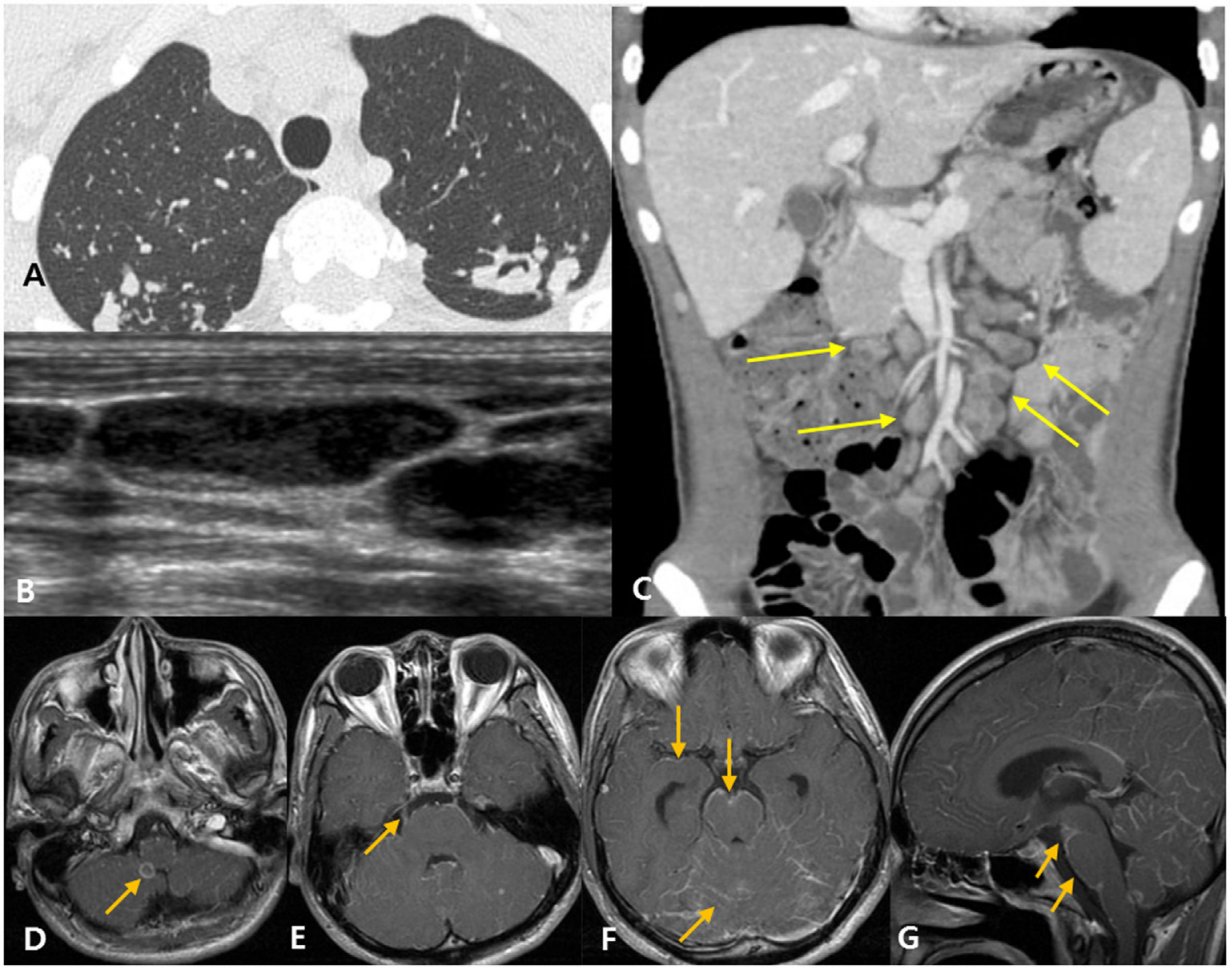
Disseminated tuberculosis case in a 22-year-old man with headache and diplopia. **(A)** The patient was diagnosed with active pulmonary tuberculosis based on chest computed tomography (CT). **(B)** Neck ultrasound shows eccentric necrosis in the enlarged lymph nodes of the neck, suggestive of tuberculosis lymphadenitis. **(C)** Abdominopelvic CT shows mesenteric lymphadenopathy (yellow arrows) and retroperitoneal lymphadenopathy (not shown), suggestive of tuberculosis lymphadenitis. **(D–G)** Post-contrast T1-weighted magnetic resonance images show rim enhancing tuberculoma [**(D)**, arrow], thick, irregular enhancement of the right trigeminal nerve [**(E)**, arrow], and linear leptomeningeal enhancement along the cerebral sulci, cerebellar folia, and the surface of the brain stem [**(F,G)**, arrows].

Of the 22 patients, three showed multiple tuberculomas. The primary origins of tuberculomas were as follows: pulmonary tuberculosis (one patient) and disseminated tuberculosis (two patients) (Figure 2). Furthermore, two patients showed both meningeal enhancement and tuberculomas (Figure 3). Thus, a total of 6 patients out of 22 showed typical findings of CNS tuberculosis.

**Figure 4:**
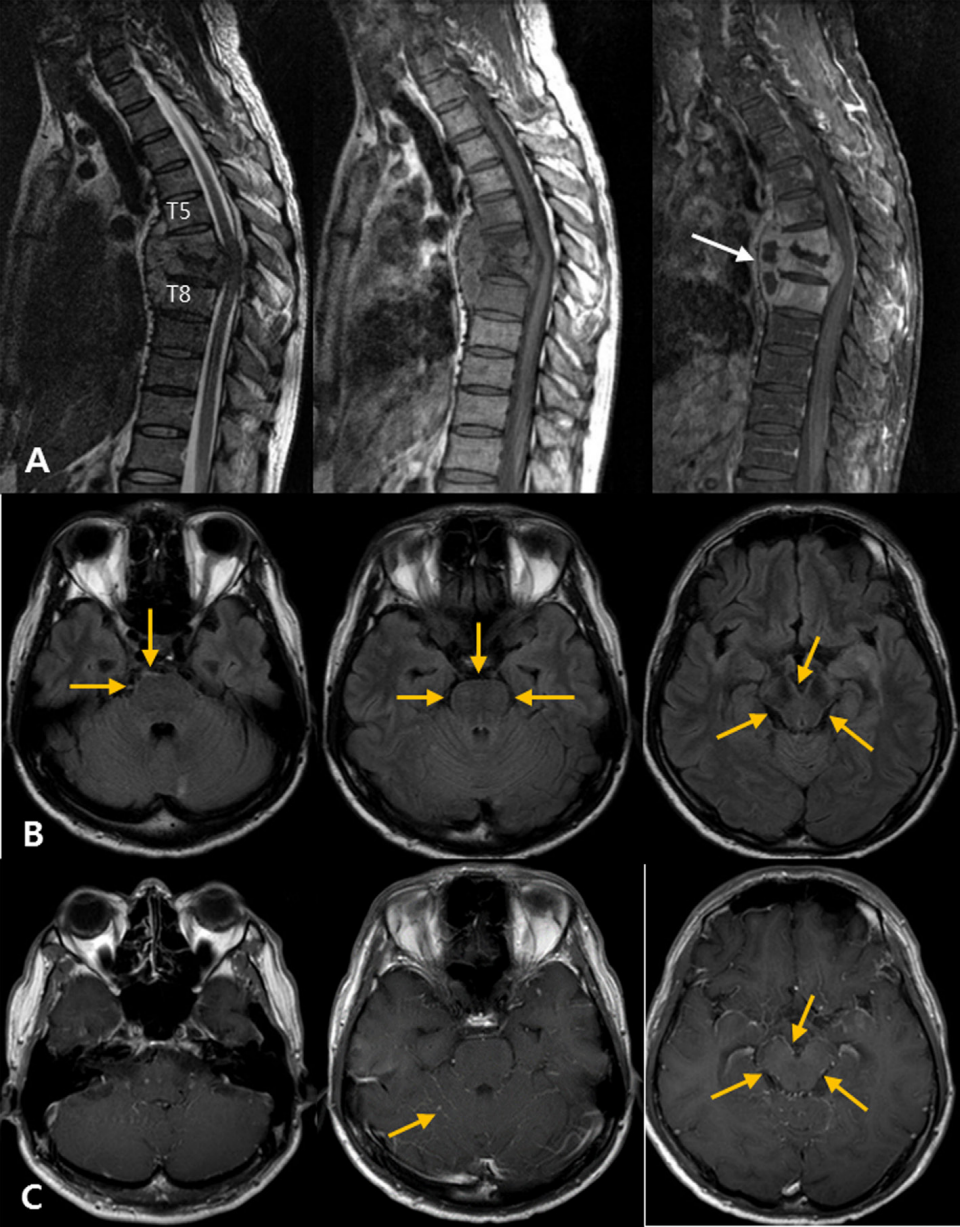
Atypical tuberculous meningitis in a 45-year-old man with low back and radiating pain. **(A)** Spine magnetic resonance (MR) shows subligamentous spreading pattern of spondylitis with T6–7 endplate erosion and paravertebral abscess formation (arrows), suggestive of tuberculous spondylitis. **(B)** One month later, the patient became mentally confused. Post-contrast T1-weighted MR images show linear high signal intensities along the pial surface of the infratentorium, including the cerebellar folia and surface of the brainstem. **(C)** Matching fluid-attenuated inversion recovery images also show subtle abnormal high signal changes along the pial surface of the infratentorial structures.

Seven patients showed atypical abnormalities on brain MR images. Five patients had abnormal high SI along the cerebellar folia. Of those five patients, two (patients No. 20 and 22) showed both typical meningitis and atypical meningitis consistently; the remaining three patients showed FLAIR abnormalities along the cerebellar folia (Figure 4).

Two patients showed mild hydrocephalus and one patient showed moderate hydrocephalus, as compared with cerebral atrophy, considering their ages (Table 1).

## Discussion

Cerebral tuberculosis occurs mainly by hematogenous spread from a primary pulmonary or extrapulmonary site or by leptomeningeal spread from a primary or secondary spinal tuberculosis, and it is more common in cases with durg-resistant tuberculosis. Well-established brain manifestations of cerebral tuberculosis are tuberculosis meningitis and tuberculomas. Tuberculous meningitis is a severe infection of the CNS, predominantly involving the brain parenchyma, meninges, and spinal cord. Leptomeningeal and basal cisternal enhancement, hydrocephalus, periventricular infarctions, and tuberculoma are well-known neuroimaging characteristics of tuberculous meningitis (12). The non-caseating and caseating tuberculomas appear iso- to high SI on both T1WI and T2WI with nodular or rim-like enhancement on post-contrast T1WI (13).

However, these typical findings are not always apparent on brain MR images. These findings may or may not appear, or may be very subtle. Therefore, early-stage cerebral tuberculosis can be overlooked if the clinician focuses on typical findings. In this study, we showed that tuberculosis can manifest other patterns of CNS infection, especially on the early stage.

The findings of tuberculous meningitis are better appreciated on MR imaging than on CT, especially on post-contrast T1WI, which show the enhancing cisternal exudates and leptomeningeal enhancement (1). Parmar et al. demonstrated that CE-FLAIR images may have a higher specificity compared to CE-T1WI for the detection of leptomeningeal enhancement (14). Furthermore, FLAIR sequence is the most important non-enhanced MR sequence to detect brain lesions. In short, early meningitis pattern can be observed on non-enhanced sequences, especially on FLAIR, as high SI along the pial surface of the infratentorium or mild hydrocephalus. Therefore, in addition to focusing on CE-T1WI, we also focus on abnormal findings in other sequences such as non-enhanced FLAIR and CE-FLAIR images.

All previous brain MR studies have focused on typical findings. If unusual findings such as abnormal SI or hydrocephalus were observed, the radiologists reported them separately, and did not correlate them with tuberculosis. Abnormal meningeal enhancement, known as the typical radiographic finding, is usually observed in the basal cisterns, Sylvian fissures, and within the sulci over the cerebral convexities (1). CNS tuberculosis develops when subependymal or subpial foci, also known as Rich foci, which are seeded during primary infection or disseminated disease, rupture and are released directly into the subarachnoid space. Then, tuberculosis bacilli become lodged in the meninges, brain, or spinal cord (1). CNS tuberculosis can manifest in a variety of forms, including tuberculous meningitis (the most common presentation), tuberculomas, tuberculous abscesses, tuberculous cerebritis, and miliary tuberculosis (1, 15). Tentorial and cerebellar meningeal enhancement are less common (16).

In this study, the most common location for unusual manifestations of brain tuberculosis, whether primary or secondary to spinal tuberculosis, was the cerebellum. This uncommon presentation can delay the diagnosis, thus leading to development of resistance to the primary treatment. MR findings include linear high SI along the cerebellar folia with apparent or subtle enhancement. These findings are most sensitively detected on FLAIR images.

The second most common neuroimaging finding was mild hydrocephalus without any brain parenchymal involvement. Hydrocephalus is a common complication of tuberculous meningitis and can be classified into two types: communicating and non-communicating (obstructive). Communicating hydrocephalus occurs more frequently than non-communicating hydrocephalus (1). Communicating hydrocephalus is a secondary imaging finding to obstruction of cerebrospinal fluid flow due to meningeal exudate (17). Non-communicating (obstructive) hydrocephalus can occur either because of outlet obstruction of the fourth ventricle by the basal exudates or mass effect of a focal parenchymal tuberculoma (16). The significance of hydrocephalus has been overlooked in cases with no brain parenchymal changes. However, clinicians should consider the possibility of disseminated brain tuberculosis, especially if ventricular size is increased, considering the patient’s age. It can be a critical point to treat the primary tuberculosis appropriately, so radiologists emphasize the importance of follow-up to clinicians.

Understanding of the pathways and mechanisms of tuberculosis dissemination could have a major impact to diagnose the early cerebral involvement. First, the hematogenous dissemination of tuberculosis from the primary site of infection, especially pulmonary alveolus, is a key step in the development of extrapulmonary tuberculosis. Trafficking of bacteria to the regional lymph nodes is essential to the development of a protective T-cell-mediated immune response, but bacteria in the bloodstream can lead extrapulmonary dissemination (18). Mycobacteria initially can cross the alveolar epithelial barrier by direct invasion and lysis of epithelial cell or by traveling within phagocytes (18). Then, the bacteria migrate from the primary site of infection to the lymphatic system and bloodstream.

The development of tuberculous spondylitis initiates through hematogenous spread *via* the epidural and perivertebral Batson plexus, which anastomoses with pleural and intercostal veins (19). Infection usually begins in the anterior part of the vertebral body adjacent to the endplate. Subsequent demineralization of the end plate allows the spread of infection to the adjacent intervertebral disk. The loose structure of the disk contributes to wide spread infection into adjacent spinal segments. As a result, more than one vertebral body, together with the intervening disks, becomes affected. This characteristic finding is known as the classic pattern of spinal involvement of tuberculosis. Infection also spreads into the paraspinal tissues, resulting in the formation of a paravertebral abscess (Pott abscess) (1).

In our study, the rate of abnormal findings on brain MRI was high among the 22 cases, because we considered the atypical findings at the FLAIR sequence. FLAIR is a highly sensitive and specific MR sequence for detecting subarachnoid space diseases, including meningitis. In meningitis, elevated protein level in cerebrospinal fluid and cellular concentrations shorten the T1 relaxation time, alter the point at which cerebrospinal fluid is nulled, and prolong the T2 relaxation time of cerebrospinal fluid. Therefore, meningitis can show as high SI along the subarachnoid space on FLAIR, as in our study. If post-contrast FLAIR is undertaken, radiologists will get more information because post-contrast T1-weighted imaging is superior to non-enhanced FLAIR in detecting meningitis. Furthermore, post-contrast FLAIR is superior to post-contrast T1WI in detecting leptomeningeal disease. However, in our study, non-enhanced FLAIR can be more useful than post-contrast T1-weighted imaging for detection of cerebral tuberculosis.

### Summary

Brain MR images in patients with cerebral tuberculosis can manifest typical findings of meningeal enhancement on the basal cistern or supratentorium and rim or nodular enhancing mass or atypical findings such as hydrocephalus and abnormal FLAIR signal changes at the infratentorium or cranial nerves.

If cerebral tuberculosis is suspected, brain MRI should be performed early. FLAIR is a highly sensitive and specific MR sequence to detect subarachnoid space diseases, including meningitis. Contrast-enhanced MR sequences such as CE-T1WI or CE-FLAIR are widely used to evaluate tuberculous meningitis.

However, because the atypical findings, such as high SI along the cerebellar folia, can manifest more apparently in non-enhanced FLAIR than CE sequence, radiologists should place a greater emphasis on FLAIR. In short, on non-enhanced FLAIR, radiologists should focus on abnormalities along the cerebellar folia and not only the typical sites of meningeal involvement.

## Ethics Statement

This retrospective study was approved by our institutional review board. Informed consent was waived because only imaging findings and demographic data were retrieved from the picture archiving and communication system (PACS) server and medical records.

## Author Contributions

JH and JP: data collection and writing the manuscript, KL: conception, design of the study, and writing the manuscript, EK, WC, H-GK, and NY: critical revision of the manuscript for important intellectual content.

## Conflict of Interest Statement

The authors declare that the research was conducted in the absence of any commercial or financial relationships that could be construed as a potential conflict of interest.
